# High-quality metagenomic-assembled genomes from sea ice and seawater of the Southern Ocean

**DOI:** 10.1128/mra.01298-24

**Published:** 2025-06-10

**Authors:** Z. M. Buthelezi, R. E. Pierneef, O. K. I. Bezuidt, T. P. Makhalanyane

**Affiliations:** 1 Department of Biochemistry, Genetics and Microbiology, DSI/NRF South African Research Chair in Marine Microbiomics, University of Pretoria 56410, Pretoria, South Africa; 2 The School for Data Science and Computational Thinking, Stellenbosch University 26697, Stellenbosch, South Africa; 3 Department of Microbiology, Faculty of Science, Stellenbosch University 98826, Stellenbosch, South Africa; California State University San Marcos, San Marcos, California, USA

**Keywords:** Southern Ocean, seawater, sea ice, dimethylsulfoniopropionate, methane monooxygenase

## Abstract

We provide high-quality metagenome-assembled genomes (MAGs) derived from seawater and sea ice samples collected in the Southern Ocean. Several MAGs encode genes associated with dimethylsulfoniopropionate (DMSP) lyase activity and methane oxidation. This resource provides insights regarding the role of microbial communities in the production of key volatile compounds.

## ANNOUNCEMENT

The Southern Ocean (SO) supports essential ecosystem services, and there is strong evidence showing the central role of microbes as mediators of these processes (1–4). These microorganisms are subject to significant environmental shifts, particularly during winter, as SO waters transition to ice-covered states (5). Here, we report the taxonomic classification and functional annotation of seven high-quality MAGs obtained from seawater and sea ice. Notably, some of these MAGs harbor functional genes linked to DMSP and methane metabolism.

Two surface seawater and two ice-core samples were collected during the Southern oCean seAsonaL Experiment (SCALE) winter cruise (July 11–31, 2022) aboard the *RV SA Agulhas II*. Seawater samples (20 L each) were obtained using the underway system and Niskin water bottles. Floating sea ice was collected using a net deployed from the ship’s aft crane. Ice cores were then sampled and stored at 4°C for thawing, resulting in 2 L of meltwater per core. These samples were filtered through 142 mm diameter isopore membrane filters (Merck, USA) to target the bacterioplankton size fraction, as previously described (6). DNA was extracted from these filters using the DNeasy PowerSoil Pro Kit (Qiagen, USA), following the manufacturer’s protocol (7). DNA quality was verified using the Qubit dsDNA Assay kit on a Qubit 4 Fluorometer (Thermo Fisher Scientific, USA), followed by 1% gel electrophoresis (8). Library preparation was performed for the four samples using the KAPA HyperPrep Kit (Roche, Switzerland) with a low-cycle PCR as detailed in the manufacturer’s protocol. Library quality and quantity were assessed using the Qubit 2.0 DNA High Sensitivity Assay (ThermoFisher, USA), Tapestation High Sensitivity D1000 Assay (Agilent Technologies, USA), and QuantStudio 5 System (Applied Biosystems, USA). Illumina 8-nt dual indices (Illumina, USA) were used for indexing and barcoding. Equimolar pooling of libraries was performed, based on QC values, prior to sequencing. Metagenomic sequencing was performed using the 2 × 150 bp sequencing chemistry NovaSeq platform (Illumina, USA), which was outsourced to Admera Health Biopharma Services, New Jersey, USA.

All analyses were performed using default parameters unless specified otherwise. The quality of raw reads was assessed using FastQC v1.15 (https://github.com/s-andrews/FastQC) (9). Low-quality reads and adapters were filtered using Trimmomatic v0.36 (10). Quality-filtered paired-end reads were assembled with metaSPAdes v3.15.5 (11), and assembly quality was evaluated using MetaQUAST v5.2.0 (12). Contigs ≥ 1,500 bp were used to reconstruct MAGs, by mapping quality-filtered reads to the assemblies using BBMap v38.95 (13). MetaBAT 2 v2.15 was used to generate bins as described previously (14), and the quality of these MAGs was assessed with CheckM v2 (15). Based on established criteria (16), seven high-quality MAGs (Table 1) were obtained and used for taxonomic classification using GTDB-TK v2.3.0 (17). These high-quality genomes are publicly available, and their annotation was carried out using the NCBI’s Prokaryotic Genome Annotation Pipeline (PGAP) (18). Identification of protein-coding genes for DMSP lyases and methane oxidation (19) was performed using eggNOG-mapper v2 (20) with DIAMOND v2.1.8 (21). The genome information, including isolation sources for the seven genomes, is summarized in Table 1.

**TABLE 1:** Information and quality report of seven MAGs together with four SRA accession numbers of their metagenomes

Genome ID	Genome accession no.	Organism	SRA accession	No. of Illumina reads	Source	Latitude (S)	Longitude (E)	Completeness (%)	Contamination (%)	Total length (bp)	GC content (%)	Total genes	Coding genes	tRNAs	ncRNAs	No. of contigs	Contig N50
OD-2 DCM_metabat.8	JBIYRN000000000	*Oceanospirillaceae bacterium*	SRX26916982	37,710,320	Deep chlorophyll maximum	58° 4	0° 6 4	99.54	0.13	2,247,091	45	2216	2159	41	4	163	26 kb
OD-2 DCM_metabat.25	JBIYRM000000000	*Cellvibrionales bacterium*	SRX26916982	37,710,320	Deep chlorophyll maximum	58° 4	0° 64	97.29	4.16	2,855,154	52	2659	2617	24	4	220	23.5 kb
OD-2 DCM_metabat.39	JBIYRL000000000	*Halioglobus sp*.	SRX26916982	37,710,320	Deep chlorophyll maximum	58° 4	0° 64	100	1.6	3,167,572	52	3051	3001	27	4	313	15.6 kb
IO-Ice_metabat.12	JBIYRP000000000	*Pseudoalteromonas sp*	SRX26916981	26,649,967	Sea ice	59° 41	0° 64	100	0.05	4,422,849	39	3942	3850	47	4	67	132.9 kb
IO-Ice_metabat.13	JBIYRO000000000	*Polaribacter sp*.	SRX26916981	26,649,967	Sea ice	59° 41	0° 64	97.73	1.01	2,506,219	35	2281	2240	32	4	214	19.4 kb
OD-2 ICE_metabat.1	JBIYRK000000000	*Flavobacteriales bacterium*	SRX26916983	38,490,889	Sea ice	58° 4	0° 64	96.62	1.69	3,049,849	32	2631	2593	30	3	15	355.5 kb
PUZ-DCM_metabat.5	JBIYRJ000000000	*Opitutales bacterium*	SRX26916984	27,225,834	Deep chlorophyll maximum	59° 99	0° 06	93.64	2.77	4,011,623	50	3785	3682	34	3	570	9.4 kb

## Data Availability

Whole genome shotgun data were deposited at DDBJ/ENA/GenBank under the BioProject accession PRJNA1167212 for assemblies and PRJNA1192238 for SRA submission. The BioSample accessions for the raw reads and genomes are listed in Table 1. EggNOG annotations for the MAGs are accessible through figshare: https://doi.org/10.6084/m9.figshare.28430819.

## References

[1] Brandt A, Gooday AJ, Brandão SN, Brix S, Brökeland W, Cedhagen T, Choudhury M, Cornelius N, Danis B, De Mesel I, Diaz RJ, Gillan DC, Ebbe B, Howe JA, Janussen D, Kaiser S, Linse K, Malyutina M, Pawlowski J, Raupach M, Vanreusel A. 2007. First insights into the biodiversity and biogeography of the Southern Ocean deep sea. Nature 447:307–311. doi:10.1038/nature05827 17507981

[2] Treusch AH, Vergin KL, Finlay LA, Donatz MG, Burton RM, Carlson CA, Giovannoni SJ. 2009. Seasonality and vertical structure of microbial communities in an ocean gyre. ISME J 3:1148–1163. doi:10.1038/ismej.2009.60 19494846

[3] Hutchins DA, Fu F. 2017. Microorganisms and ocean global change. Nat Microbiol 2:17058. doi:10.1038/nmicrobiol.2017.58 28540925

[4] Wilkins D, van Sebille E, Rintoul SR, Lauro FM, Cavicchioli R. 2013. Advection shapes Southern Ocean microbial assemblages independent of distance and environment effects. Nat Commun 4:2457. doi:10.1038/ncomms3457 24036630

[5] Abernathey RP, Cerovecki I, Holland PR, Newsom E, Mazloff M, Talley LD. 2016. Water-mass transformation by sea ice in the upper branch of the Southern Ocean overturning. Nature Geosci 9:596–601. doi:10.1038/ngeo2749

[6] Pesant S, Not F, Picheral M, Kandels-Lewis S, Le Bescot N, Gorsky G, Iudicone D, Karsenti E, Speich S, Troublé R, Dimier C, Searson S, Tara Oceans Consortium Coordinators. 2015. Open science resources for the discovery and analysis of Tara Oceans data. Sci Data 2:150023. doi:10.1101/019117 26029378 PMC4443879

[7] Weber L, DeForce E, Apprill A. 2017. Optimization of DNA extraction for advancing coral microbiota investigations. Microbiome 5:18. doi:10.1186/s40168-017-0229-y 28179023 PMC5299696

[8] Nakayama Y, Yamaguchi H, Einaga N, Esumi M. 2016. Pitfalls of DNA quantification using DNA-binding fluorescent dyes and suggested solutions. PLoS One 11:e0150528. doi:10.1371/journal.pone.0150528 26937682 PMC4777359

[9] Brown J, Pirrung M, McCue LA. 2017. FQC Dashboard: integrates FastQC results into a web-based, interactive, and extensible FASTQ quality control tool. Bioinformatics 33:3137–3139. doi:10.1093/bioinformatics/btx373 28605449 PMC5870778

[10] Bolger AM, Lohse M, Usadel B. 2014. Trimmomatic: a flexible trimmer for Illumina sequence data. Bioinformatics 30:2114–2120. doi:10.1093/bioinformatics/btu170 24695404 PMC4103590

[11] Nurk S, Meleshko D, Korobeynikov A, Pevzner PA. 2017. metaSPAdes: a new versatile metagenomic assembler. Genome Res 27:824–834. doi:10.1101/gr.213959.116 28298430 PMC5411777

[12] Mikheenko A, Saveliev V, Gurevich A. 2016. MetaQUAST: evaluation of metagenome assemblies. Bioinformatics 32:1088–1090. doi:10.1093/bioinformatics/btv697 26614127

[13] Bushnell B. 2014. BBMap: a fast, accurate, splice-aware aligner

[14] Kang DD, Li F, Kirton E, Thomas A, Egan R, An H, Wang Z. 2019. MetaBAT 2: an adaptive binning algorithm for robust and efficient genome reconstruction from metagenome assemblies. PeerJ 7:e7359. doi:10.7717/peerj.7359 31388474 PMC6662567

[15] Chklovski A, Parks DH, Woodcroft BJ, Tyson GW. 2023. CheckM2: a rapid, scalable and accurate tool for assessing microbial genome quality using machine learning. Nat Methods 20:1203–1212. doi:10.1038/s41592-023-01940-w 37500759

[16] Bowers RM, Kyrpides NC, Stepanauskas R, Harmon-Smith M, Doud D, Reddy TBK, Schulz F, Jarett J, Rivers AR, Eloe-Fadrosh EA, et al.. 2017. Minimum information about a single amplified genome (MISAG) and a metagenome-assembled genome (MIMAG) of bacteria and archaea. Nat Biotechnol 35:725–731. doi:10.1038/nbt.3893 28787424 PMC6436528

[17] Chaumeil P-A, Mussig AJ, Hugenholtz P, Parks DH. 2020. Oxford University Press.

[18] Tatusova T, DiCuccio M, Badretdin A, Chetvernin V, Nawrocki EP, Zaslavsky L, Lomsadze A, Pruitt KD, Borodovsky M, Ostell J. 2016. NCBI prokaryotic genome annotation pipeline. Nucleic Acids Res 44:6614–6624. doi:10.1093/nar/gkw569 27342282 PMC5001611

[19] Buchfink B, Reuter K, Drost H-G. 2021. Sensitive protein alignments at tree-of-life scale using DIAMOND. Nat Methods 18:366–368. doi:10.1038/s41592-021-01101-x 33828273 PMC8026399

[20] Cantalapiedra CP, Hernández-Plaza A, Letunic I, Bork P, Huerta-Cepas J. 2021. eggNOG-mapper v2: functional annotation, orthology assignments, and domain prediction at the metagenomic scale. Mol Biol Evol 38:5825–5829. doi:10.1093/molbev/msab293 34597405 PMC8662613

[21] Buchfink B, Xie C, Huson DH. 2015. Fast and sensitive protein alignment using DIAMOND. Nat Methods 12:59–60. doi:10.1038/nmeth.3176 25402007

